# Microneedling With RF‐Assisted Skin Penetration Improves the Hard‐to‐Treat Periorbital Wrinkles: Nonrandomized Clinical Trial

**DOI:** 10.1111/jocd.16559

**Published:** 2024-10-17

**Authors:** Dorina Cheles, Yuri Vinshtok, Amikam Gershonowitz

**Affiliations:** ^1^ DC Ophthalmology & Aesthetic Medicine Yokneam Israel; ^2^ Pollogen Ltd Tel Aviv Israel

**Keywords:** aging face, Divine Pro, periorobital wrinkles, RF microneedling, voluderm

## Abstract

**Introduction:**

This prospective study evaluates the efficacy and safety of minimally invasive radiofrequency microneedling (RFMN) for the correction of periorbital wrinkles. This study aimed to address the challenges posed by the periorbital region's unique anatomy and the limitations of existing non‐surgical treatments.

**Methods:**

Twenty‐four subjects, ranging from 34 to 54 years old with Fitzpatrick skin types II–V, underwent a series of treatments using the Voluderm RF microneedling device. Participants were divided into two groups based on the severity of their wrinkles and treated with customized protocols over four sessions, with a 3‐month follow‐up to assess outcomes. Efficacy was determined through comparisons of pre‐treatment and post‐treatment wrinkle severity, using the Lemperle Classification of Facial Wrinkles (LFW) and the Global Aesthetic Improvement Scale, evaluated by both the investigators and the patients. Safety and tolerability were assessed through adverse event reporting and a Visual Analog Scale for treatment discomfort.

**Results:**

The results demonstrated a statistically significant reduction in periorbital wrinkle severity, with an average 49% decrease of LFW score and improvements noted across all skin types in both groups. The aggregated facial LFW score decreased from baseline mean 13.00 ± 4.75 to 6.09 ± 3.90 (*p* < 0.05). The treatment was well tolerated without anesthetics, with minimal downtime and few adverse events, which were transient and resolved without intervention.

**Conclusion:**

The efficacy of Voluderm RF microneedling in improving periorbital wrinkles of variable severity was demonstrated in patients with diverse skin types. The unique RF‐assisted mechanism of the skin penetration granted a high tolerability of the treatments, negligible downtime, and minimum number of adverse events with self‐resolution.

## 1 Introduction

Skin wrinkles in the lateral external canthus and lower eyelid areas, commonly known as periorbital wrinkles (POWs), have a great social impact as the earliest perception of facial aging. Environmental oxidative stress, reduction of cutaneous microcirculation, and structural loss of the dermal elements advance formation of the skin wrinkling [1]. Resorption of the inferolateral orbit bone and repetitive contraction of the eye‐moving musculature additionally contribute to progressive worsening, from superficial lines to deep coarse wrinkles [2]. Age‐related decline in collagen and elastin production, exposure to solar ultraviolet grant thinning and decreased elasticity of the skin toward the formation and gradual deepening of wrinkles [3]. The process is dynamic, and the severity is influenced by ongoing atrophy and depletion of the soft tissue volume.

Non‐surgical correction of POWs is challenging due to proximity of the crucial anatomical structures. Dermal fillers and neurotoxins provide short‐living effects and for inexperienced injectors are associated with risks of asymmetry, persistent edema, nodules, and severe hematoma [4]. Effects of chemical peels are encouraging but limited to light or medium peeling agents with questionable penetration through the stratum corneum [5]. High efficacy of ablative laser resurfacing is derogated by the associated pain, long‐lasting recovery, and complications [6]. Non‐ablative lasers offer more tolerable treatments but are linked to persistent skin peeling [7].

Minimally invasive radiofrequency microneedling (RFMN) provides an effective alternative. RFMN implements an array of metal microelectrodes mechanically penetrating the skin with subsequent emission of RF pulse and heating the surrounding dermal tissue to a temperature above 65°C. In the early developed monopolar configuration, electrical current flows from the microneedle to a grounding pad located on the body, causing pain during the procedure [8]. A widely used bipolar configuration generates electrical current between the microneedles. The thermal impact triggers the wound‐healing mechanism, which results in the regeneration of extracellular matrix. Clinical studies demonstrated photographic improvement and decreased severity of the facial wrinkles treated with bipolar RFMN [8, 9]. However, the treatments are associated with bleeding and pain and require a lengthy pre‐procedural anesthesia [10]. Adverse incidents of swelling, paresthesia, eyelid tremor, and post‐inflammatory hyperpigmentation (PIH) in higher Fitzpatrick skin types take a long time to resolve [8].

The Voluderm RF microneedling (Pollogen, Israel) reduces shortages of bipolar microneedling by implementing a distinctive configuration and unique mechanism for the RF‐assisted skin penetration. The electrical current is generated between the array of the non‐insulated microelectrodes and the corresponding return micro‐plates. The microelectrodes deliver RF in a sequence: first to ablate and penetrate the stratum corneum and then to apply it to the dermis. As a result, RF creates multiple zones of fractional ablation, coagulation and spreads the generated heat through the surrounding tissue. Adjustment of the RF pulse power modulates the extent of coagulation, while the pulse duration is directly proportioned to the depth of ablation. Histology studies verified that at low settings, Voluderm impact results in shallower ablation and smaller coagulation than at the higher energy settings [11]. The triggered wound healing was manifested with lymphatic infiltrate, increased epidermal hyaluronan and mitotic index, and eventually led to fibrosing granulation and epidermal re‐epithelization [12]. The treatments were shown to improve skin laxity and wrinkles in the middle and lower face at low rate of adverse incidents and negligible downtime [11, 13]. We rationalized that easily tolerable Voluderm treatments could be effectively implemented for correction of the hard‐to‐treat periorbital wrinkles. A nonrandomized clinical study investigated the treatment response in periorbital wrinkles of variable severity.

## 2 Materials and Methods

A prospective, single‐center study was conducted in accordance with the ethical principles set out in the Declaration of Helsinki 2000. Male and female candidates with shallow‐to‐deep POWs were enrolled in the study and signed consent forms including photo‐publishing permission. Pregnant or lactating females, patients with history of keloids, active infectious or inflammatory skin conditions, recent filler/neurotoxins injections, ablative skin resurfacing, lifting surgery, isotretinoin, or retinoids therapy were excluded.

The study objectives include efficacy and safety of the Voluderm treatments toward improvement of the superficial and deep periorbital wrinkles. Severity of the wrinkles was determined by comparison to the reference photo‐numeric scale (Lemperle Classification of Facial Wrinkles, LFW): (0) no wrinkles, (1) just perceptible, (2) shallow, (3) moderately deep wrinkle, (4) deep with well‐defined edges, and (5) very deep or redundant fold [14]. Participants were divided into two treatment groups: a group with superficial wrinkles (LFW class 1–2) and a group with deep wrinkles (LWF class 3–4). Folds and very deep wrinkles were excluded from the treatments.

**FIGURE 1:**
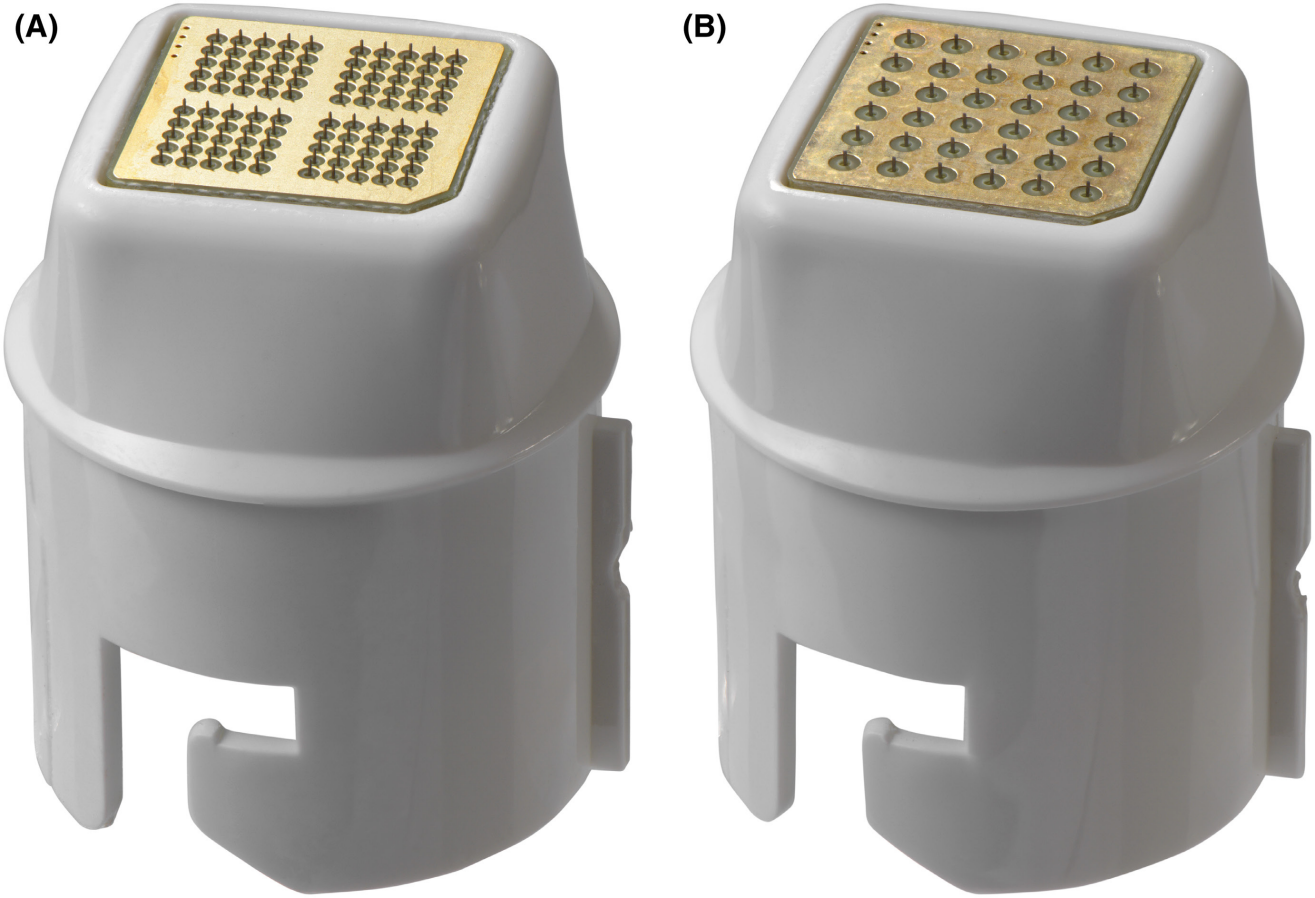
Voluderm tips utilized in the study: (A) Gen100 depicting 100 microneedle electrodes, (B) Gen36L depicting 36 microneedle electrodes.

Each group was treated with corresponding disposable Voluderm tip: Gen100 for superficial wrinkles and Gen36L for deeper wrinkles (both by Divine Pro, Pollogen, Israel). Microelectrode matrixes of the Gen100 and Gen36L tips contain respective 100 or 36 non‐insulated ultra‐thing microelectrodes and produce either 100 or 36 entry points over 1 cm^2^ skin area at each pass. At 1.0 mm length, Gen36L microelectrodes generate a deeper thermal impact than Gen100 with 0.6 mm length (Figure 1).

Participants in each group received four treatments at 2‐week intervals and were reassessed 3 months after the last treatment. The data collected at each visit were stratified for analysis between the Gen100 and Gen36L groups. Safety data were collected throughout the entire study period.

### 2.1 Treatment Protocol

After obtaining a written informed consent, the skin was cleansed. A single pass of Voluderm RFMN was applied to the entire face and the wrinkles of the most patient's concerns. POWs areas were treated more intensively, with triple passes applied as the crisscross stocking applications. The maneuver increased density of the stimulation impact without missing the position. When treating lateral orbital wrinkles, the skin was stretched lateral‐and‐out over zygomatic bone to provide safe access to the bottom of the wrinkle invagination. For the wrinkles in the infraorbital region, the passes were applied to the skin pulled down along the infraorbital bone margin.

The treatment parameters started at a more conservative level and were gradually increased under the patient tolerability. The pulse power was raised to achieve a stronger coagulation impact. The pulse duration was lowered over infraorbital notches due sensitivity and proximity of the superficial nerve.

The treatment was completed with topical 0.67% trolamine emollient to hydrate the skin and shorten downtime. Patients were advised to avoid sun exposure and wear sunscreen for 1 week thereafter.

### 2.2 Study Measurements

The treatment efficacy was assessed according to the digital clinical photographs taken before the treatment and at 3‐month follow‐up. The images were obtained under the same lighting and position provided by the 360° rotating LED light system (YN300; Yongnuo Digital, China). Post‐treatment severity of the wrinkles was compared to the baseline. Post‐treatment POW images were separately graded by the physician and patients using Global Aesthetic Improvement Scale (GAIS), comprised of “much worse”, “worse”, “no change”, “improved”, and “very much improved” [15]. Additionally, patients subjectively measured satisfaction with the treatment results on the three‐point scale: (1) “not satisfied”, (2) “satisfied”, and (3) “very satisfied”. Procedural discomfort was rated by the patients using the Visual Analog Scale (VAS) for pain assessment, from 0 (no pain) to 10 (intolerable pain). Safety of the treatments was based on the adverse events (AE) collected and evaluated throughout the study period.

The measures were quantified, and the values were summarized as mean ± standard deviation (SD). The difference between baseline and follow‐up was analyzed using *t*‐test and considered significant if *p*‐value < 0.05.

## 3 Results

Twenty‐four subjects (23 female and 1 male), age 34–54 years and Fitzpatrick skin types II–V received the Voluderm treatments; 23 patients were presented for the follow‐up assessment. Prior to the treatment, subjects demonstrated facial rhytidosis, with periorbital wrinkles as the most emotionally burdensome. The average baseline LFW score of the periorbital wrinkles was 3.1. Fifteen patients were allocated to the superficial wrinkle treatment group and nine patients to the group with deep wrinkles (Table 1).

**TABLE 1:** Demographic characteristics and allocation of the study population.

	No. patients	Superficial wrinkles group (Gen100 tip)	Deep wrinkles group (Gen36L tip)
Female	23	8	15
Male	1	1	—
Fitzpatrick type II	6	3	5
Fitzpatrick type III	5	1	4
Fitzpatrick type IV	10	4	5
Fitzpatrick type V	1	—	1

### 3.1 Overall Efficacy Results

**FIGURE 2:**
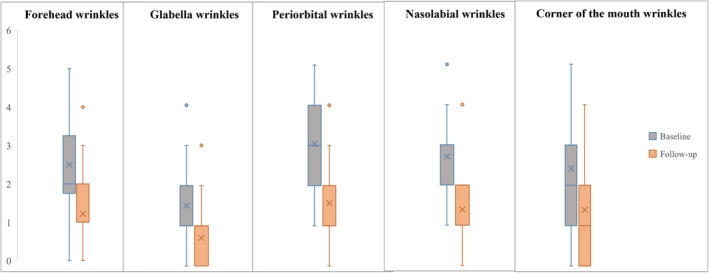
Changes in the wrinkle severity scores before and at the 3‐month follow‐up (Lemperle Classification of Facial Wrinkles, LFW) (*p* < 0.05).

Data analysis revealed a decrease in the aggregated facial LFW score 3 months after the last treatment, from baseline mean 13.00 ± 4.75 to 6.09 ± 3.90 (*p* < 0.05). The wrinkle severity was significantly lower in forehead, glabella, periorbital, nasolabial, and corner of the mouth areas (*p* < 0.05) (Figure 2).

**FIGURE 3:**
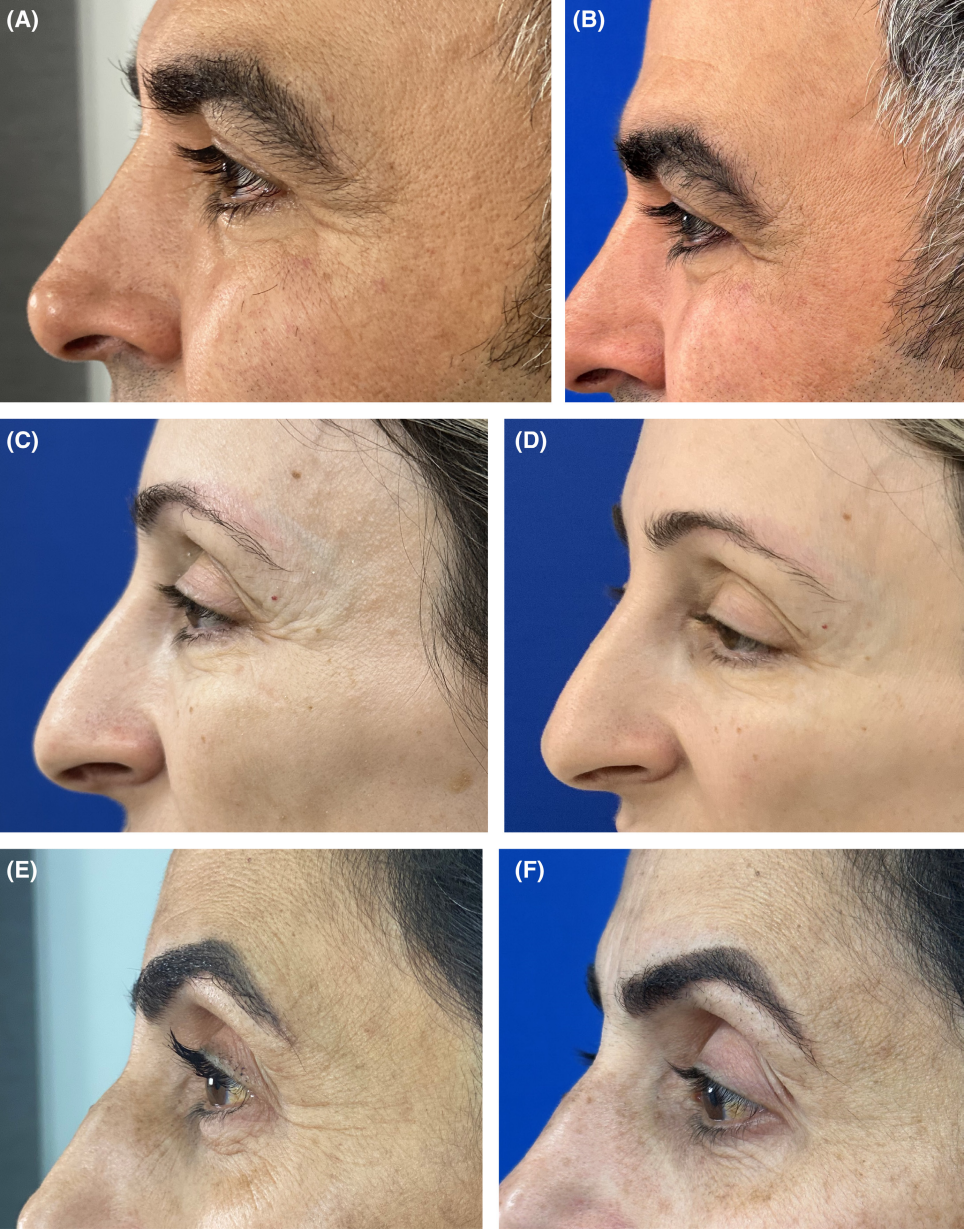
Representative “before and after” photograph shows improvement in POWs appearances: A 51‐year‐old male before (A) and after (B), a 47‐year‐old before (C) and after (D), a 48‐year‐old before (E) and after (F).

**FIGURE 4:**
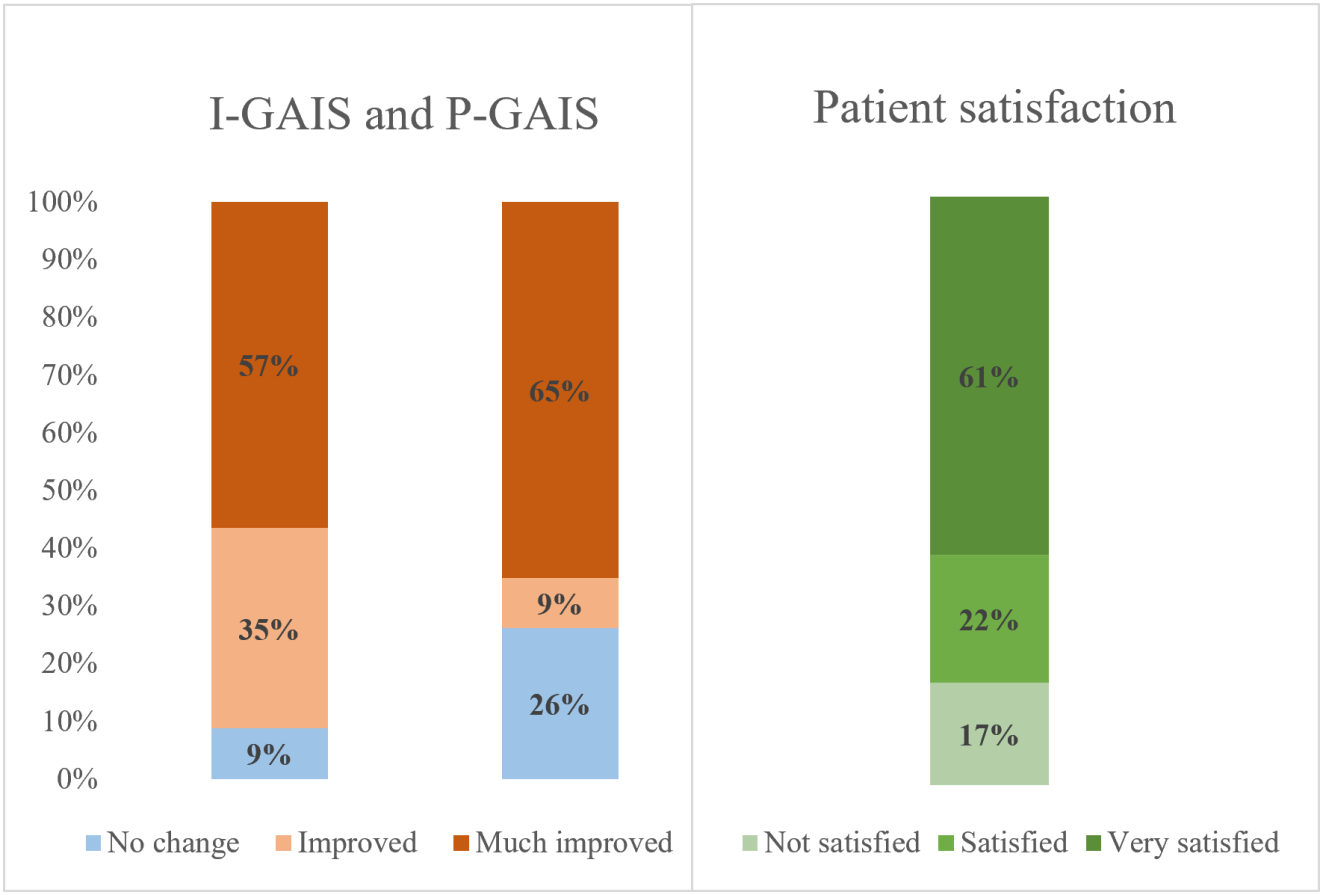
Investigator‐ and patient‐assessed Global Aesthetic Improvement Scale (I‐GAIS, P‐GAIS) and overall patient satisfaction at follow‐up. Bars represent the percentage of patients in each category.

Overall severity of the periorbital wrinkles was diminished for at least one grade in 96% of participants, with an average 49% decrease of the LFW score (*p* < 0.05). Representative “before and after” photographs showed improvement in POWs appearances (Figure 3). Photography images taken at the follow‐up were independently evaluated by the investigator (Investigator Global Aesthetic Improvement Scale, I‐GAIS) and patients (Patient Global Aesthetic Improvement Scale, P‐GAIS) by answering the GAIS questionnaire. There was a physician/patient agreement in the grade of the observed improvement: an average of 4.5 and 4.4, respectively. Patient rated POWs “improved” or “very much improved” in 71%. Eighty‐three percent of the study population reported satisfaction with the comfort, recovery time, and the treatment results (Figure 4).

### 3.2 Subgroup Efficacy Results

Both treatment groups showed reduction in LFW score: from the baseline 2.00 ± 0.46 to post‐treatment 1.00 ± 1.08 in the Gen100 group and from 3.00 ± 0.96 to 2.00 ± 1.16 in the Gen36L group (both *p* < 0.05). The treatment response was greater in the deep wrinkles compared to more superficial wrinkles. Investigator observed global improvement in 100% of the Gen36L group patients compared to 76% in the Gen100 group. However, satisfaction with the treatment results was high in both groups: 88% in the Gen100 group and 80% in the Gen36L group.

### 3.3 Safety and Tolerability

Despite transient erythema occurring after each procedure, the patients were able to return to their daily activities shortly after the treatment. Pin‐point epidermal scabs resolved in 2–3 days. Mild edema, erythema, and itching reported in four cases subsided without therapy in 3 days. A topical steroid cream was prescribed for facial edema in one case.

Treatments generated mild discomfort at the average subjective VAS 4.1, without numbing cream. The discomfort was more intense in the Gen100 group (an average 5.4 (4–7)) comparing to an average 3.4 (1–6) in the Gen36L group.

## 4 Discussion

The present study has shown the use of Voluderm RF microneedling for correction of the periorbital wrinkles. The study demonstrated that series of treatments with unique RF‐assisted mechanism of skin penetration were well tolerated, with minimal downtime or adverse events. Using arrays of short or longer RF microelectrodes the treatments significantly improved the wrinkles of variable severity.

Periorbital wrinkles appear as the earliest facial manifestation of dermal degradation. Overactivity of the *orbicularis oculi* muscles along with flattening of the dermo‐epidermal junction, thinning of the dermis, and redistribution of subcutaneous tissue result in dynamic and static skin wrinkles, volume loss, infraorbital hollows, and deepening of the superior sulcus [6].

The highly complex anatomy and proximity to the eyeballs make the periorbital area quite difficult for the corrective modalities. Aggressive CO_2_ photo‐thermolysis of the thin periorbital skin is associated with burns, hypopigmentation, and eyelid malposition [16]. Proximity of the orbicularis and frontalis muscles presents sufficient risk for neurotoxins injections and may lead to ptosis and paralytic ectropion [17]. The density of the periorbital vascular structures and proximity of zygomaticotemporal nerve links injections of dermal fillers to ischemic complications, local whitening or blueness, and visual blurring [18]. The late inflammatory reaction to HA contributes to occurrence of nodules and granulomas in the region up to several weeks thereafter [19].

Minimally invasive radiofrequency microneedling generates high heat and creates fractional zones of ablation in the dermis. By inducing bulk heating RFMN generates several thermo‐biological factors synergistically contributing to regeneration of dermal wrinkles [20]. RF‐induced ablation activates the wound‐healing mechanism and causes cellular infiltration, neovascularization, and formation of granulation tissue. Dispersion of sub‐necrotic heat results in fibroblast stimulation and subsequent collagen and elastin neo‐synthesis [21]. Healing of the Voluderm micro‐wounds revealed inflammation and granulation beginning on day 1, followed by fibrinous tissue completely filling the ablation channel and epidermal epithelization on day 14 [11].

A limited number of clinical studies, mainly on Asian population, demonstrated successful treatment of periorbital rhytids with RFMN. Jeon [22] showed increased expression of procollagen III and elastin along with improved wrinkle grading in 18 weeks after RFMN. Lee [10] reviewed digital images of 20 Korean patients and reported a decrease of the suborbital wrinkle score 6 months after the treatment. A study by Kim [20] demonstrated progressive decrease in Fitzpatrick score of the static periorbital wrinkles up to 6 months after the three‐treatment course.

In the current study, we used non‐insulted Voluderm RF microneedles with unique RF‐assisted mechanism for skin penetration. We achieved sufficient decrease in rhytids in the periorbital area, generally considered as a “hard‐to‐treat” anatomical region. The treatments focused on the lateral canthal (crow's feet) and the low lid wrinkles. Post‐treatment photo‐numeric assessment revealed reduction of the wrinkles grade, which indicated beneficial decrease of condition severity. The treatments were performed using Voluderm tips with two different lengths, depending on the wrinkle depth type, superficial or deep. Post‐treatment decrease of the wrinkle severity was statistically significant in both treatment groups.

Reasoning for the microelectrode length was justified by the histopathology rationales. Formation of periorbital rhytids is associated with alteration of epidermis–dermis junction (EDJ), an important component of the skin‐supportive structure [23]. Microelectrodes in both Voluderm tips are long enough to easily reach anatomically superficial EDJ with hyperthermia and mechanical stress. As a response, it provides stimulation to neighboring fibroblasts and caveolin‐1, a crucial scaffolding protein associated with intercellular adhesion and extracellular matrix organization [24].

**FIGURE 5:**
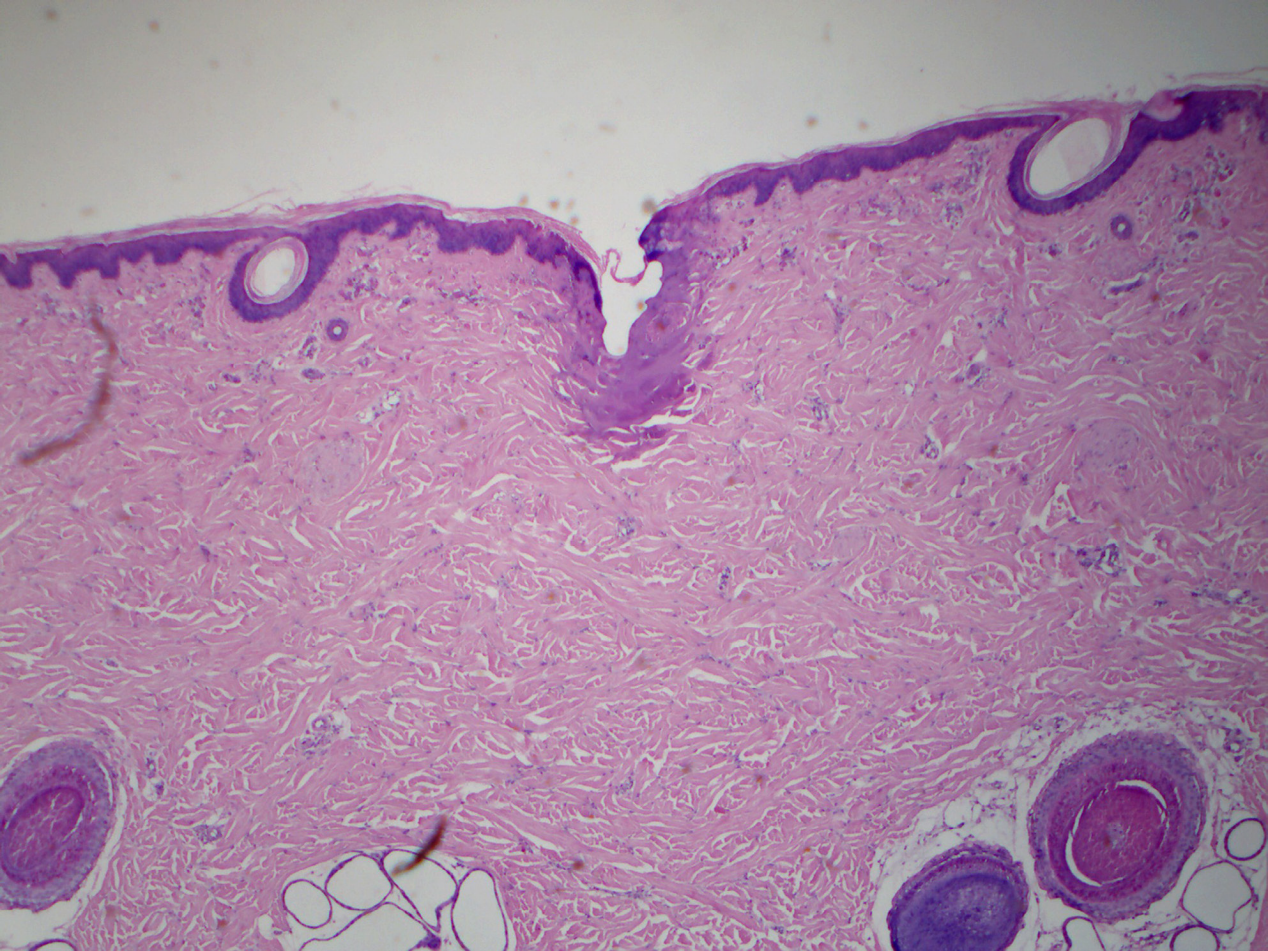
Skin histology sample of the Voluderm micro‐wound: Fractional zones of ablation in epidermis and dermis, coagulative necrosis in the papillary and upper reticular dermis. Normal skin areas are visible adjacent to the affected areas (H&E stain, magnification: 320). Skin sample: Porcine abdomen skin treated in vivo with Voluderm Gen100 tip. Immediately following treatment, a collected biopsy was fixed in 4% formaldehyde solution for 48 h. The sample was embedded and sliced exposing the central part of the block. The slides were stained with H&E and scanned for the ablation and coagulation zones (Courtesy of Dr. A. Gershonowitz, Pollogen).

Histological studies show that thermal injury extends the length of RF microneedles [9, 25]. Non‐insulated RFMN generates dispersion of the heat downward and sidewise from the ablation channel. In the Voluderm study of Kauvar [11], depth and diameter of the residual coagulation exceeded the actual size of the microelectrode by 20% and 100%, respectively (Figure 5). Furthermore, the gross anatomy study of lateral canthal wrinkles [26] revealed that shallow wrinkles progressively decrease thickness of its dermis to one‐half of the original height. As the wrinkle becomes deeper, the dermis stops thinning and curves down into the subcutaneous layer. Therefore, we rationalized that the wrinkled periorbital dermis can be efficiently accessible with either the 0.6‐mm Gen100 or the 1.0‐mm Gen36L microneedles.

The study indicated efficacy of Voluderm in treatment of periorbital wrinkles, regardless of the Fitzpatrick skin type. LFW grades decreased by 52% in type II, by 48% in type III, by 50% in type IV, and by 20% in type V skin.

Contrary to lasers with their selective photothermal effect on dermal chromophores, RF energy has a generalized electrothermal effect and can be used in all skin types [27]. Still, darker‐skinned patients run a greater risk of developing hyperpigmentation following RFMN treatments. Despite preemptive use of melanogenesis inhibitors, the reported 10%–13% PIH rate is believed to relate to high energy required for bipolar RFMN to achieve sufficient efficacy [10]. Occurred epidermal ablation and deeper coagulation lead to adverse pigmentary changes, which was vastly reduced when using Voluderm.

No PIH occurred among the study subgroup with high Fitzpatrick skin types IV–V (46% of the study population). With Voluderm RF‐assisted penetration, the injury to the epidermis is minimal and does not lead to undesired hyper‐melanosis. Additionally, the Voluderm mechanism provides an overlap of the energy emission surrounding the neighbor microelectrodes. Accordingly, the density of the RF impact on the skin between the microelectrodes is doubled without need for high energy.

Treatment downtime was accompanied by mild erythema and tiny scabs usually resolved in 2–3 days. Out of 84 performed Voluderm treatments, four incidents of moderate edema and itching required attention; however, the rate and severity were insignificant, compared to other RFMN devices [8, 9, 10, 11].

Treatments were tolerated well without administering pre‐treatment anesthetics. Exposure of the periorbital skin to the triple Voluderm passes, as required to increase density of the RF impact, was safe. Overall procedural discomfort measured by VAS was mild and slightly higher in the Gen100 group, compared to the Gen36L group (VAS 5.4 vs. VAS 3.4, respectively). The difference is due to the sensitivity toward the higher number of Gen100 microelectrodes in contact with the skin.

Non‐proportional size of the current subgroups may question the comparison; however, the achieved rate of improvement was statistically significant in both groups. The study focus was limited to correction of the wrinkles and did not address other signs of periorbital aging, including fat herniation, dyspigmentation, and infraorbital hollows. Further trials should investigate Voluderm for curing these comorbidities. It was not a controlled study due to a valid concern that the split‐face controlled design could lead to a high drop‐off rate.

## 5 Conclusion

The efficacy of Voluderm RF microneedling in improving periorbital wrinkles of variable severity was demonstrated in patients with diverse skin types. The unique RF‐assisted mechanism of the skin penetration granted a high tolerability of the treatments, negligible downtime, and minimum number of adverse events with self‐resolution.

## Author Contributions

Dorina Cheles performed the treatment procedures and analyzed the data. Yuri Vinshtok developed treatment protocol and analyzed the data. Amikam Gershonowitz provided technology consultancy. All authors had full access to all of the data in this study and took complete responsibility for the integrity of the data and the accuracy of the data analysis.

## Ethics Statement

The authors confirm that the ethical policies of the journal, as noted on the journal's author guidelines page, have been adhered to. A signed informed consent for the treatment procedures and publishing (including images and data) was obtained from all participants in the current investigation prior to the treatments. Collection and management of the patient data were conducted in accordance with the ethical guidelines and principles of the 1975 Declaration of Helsinki.

## Conflicts of Interest

The authors declare no conflicts of interest.

## Data Availability

The data that support the findings of this study are available on request from the corresponding author. The data are not publicly available due to privacy or ethical restrictions.
